# Rare type A lymphomatoid papulosis initially presenting as a giant ulcer: a case report and literature review

**DOI:** 10.3389/fimmu.2025.1606027

**Published:** 2025-07-01

**Authors:** Xiangru Chen, Qingling Zhang, Jingtong Zhao, Lin An, Yuxi Jia

**Affiliations:** ^1^ Department of Dermatology, China-Japan Union Hospital of Jilin University, Changchun, Jilin, China; ^2^ Department of Breast Surgery, China-Japan Union Hospital of Jilin University, Changchun, Jilin, China

**Keywords:** lymphomatoid papulosis, keratoacanthoma, giant ulcer, pseudoepitheliomatous hyperplasia, nodules

## Abstract

**Background:**

Lymphomatoid papulosis (LyP) is a rare, chronic, recurrent, self-healing, indolent cutaneous lymphoproliferative disorder. Histologically, it resembles malignant lymphoma; however, its clinical manifestations are predominantly characterized by benign behaviors, including recurrent papules, nodules, and necrotic lesions.

**Case presentation:**

We report a case of a middle-aged female who initially presented with a giant ulcer on the right foot and was surgically treated at another hospital as a keratoacanthoma (KA). Over subsequent months, she developed scattered papules and nodules on the trunk and limbs. A comprehensive clinical and histopathological reassessment confirmed a diagnosis of LyP Type A. Notably, the initial ulcerative lesion represented an atypical feature of LyP rather than a conventional KA. Finally, the patient was successfully treated with methotrexate and interferon, resulting in complete resolution of the skin lesions without recurrence.

**Conclusions:**

In summary, this case highlights that a giant ulcer exhibiting pseudoepitheliomatous hyperplasia (PEH) in histopathology may indicate LyP. Careful assessment for atypical lymphocytic infiltration and further immunohistochemical evaluation are essential for accurate diagnosis. When single clinical or histopathological findings are insufficient to provide a comprehensive understanding of the disease, thorough evaluation and dynamic monitoring are critical for diagnosing and managing complex cases.

## Introduction

LyP is a rare, chronic, recurrent, self-healing, low-grade malignant cutaneous lymphoma. Histologically, it exhibits features resembling those of malignant lymphoma and clinically manifests as a self-healing, recurrent disease characterized by papules, nodules, and necrosis (1, 2). In addition to these typical manifestations, case reports have also described LyP associated with squamous cell carcinoma (SCC)-like or KA-like skin changes (3–5). The disease was first described in 1965 by Dupont in Germany and subsequently named LyP by Macaulay in 1968 (1). LyP and primary cutaneous anaplastic large-cell lymphoma (C-ALCL) both belong to the category of cutaneous CD30+ T-cell lymphoproliferative disorders and exhibit highly similar histological features and immunophenotypes.

We report the case of a 57-year-old Chinese woman with LyP Type A, who initially presented with a large ulcer on the dorsum of her right foot, followed by the subsequent development of scattered papules and nodules on the trunk and limbs. Pathological examination of the ulcer on the dorsum of the foot revealed PEH, which had been previously misdiagnosed as KA. Following a comprehensive evaluation of the clinical manifestations and relevant pathological examinations, the patient was ultimately diagnosed with LyP. The KA-like changes were determined to be reactive epidermal hyperplasia, which was a part of the disease evolution. Atypical initial clinical and pathological presentations significantly increase the likelihood of misdiagnosis, underscoring the diagnostic challenges inherent in LyP.

## Case presentation

The patient was a 57-year-old Chinese woman who presented to our dermatology clinic in February 2024. Four months prior, she had developed an ulcer on the dorsum of her right foot, measuring approximately 3.0 cm × 4.0 cm, with elevated margins. Without seeking medical attention, the lesion gradually increased in size and failed to heal. Two months prior, the patient visited the department of Hand and Foot Surgery at Hospital A. Upon admission, she underwent extensive excision of the skin lesion followed by rotational flap suturing. The excised tissue was sent for pathological examination, which resulted in a diagnosis of KA. Postoperatively, the surgical wounds healed well. During her hospital stay, the attending physician identified scattered dark purple-red nodules on the patient’s trunk and both upper limbs. Immediately following discharge, the patient visited the Department of Dermatology at Hospital B, where separate pathological examinations were performed on the lesions located on the back and right forearm; However, no definitive diagnosis was established. She was administered a trial of topical corticosteroid therapy, But the condition persisted and the number of nodules gradually increased. Consequently, the patient sought further evaluation and treatment at the dermatology department of the China-Japan Union Hospital of Jilin University. During the disease course, the patient denied systemic symptoms (e.g., fatigue, weight loss). There was no history of infectious diseases, relevant family history, or personal cancer history. She denied a history of trauma, insect bites, or use of irritating topical medications.

**Figure 1:**
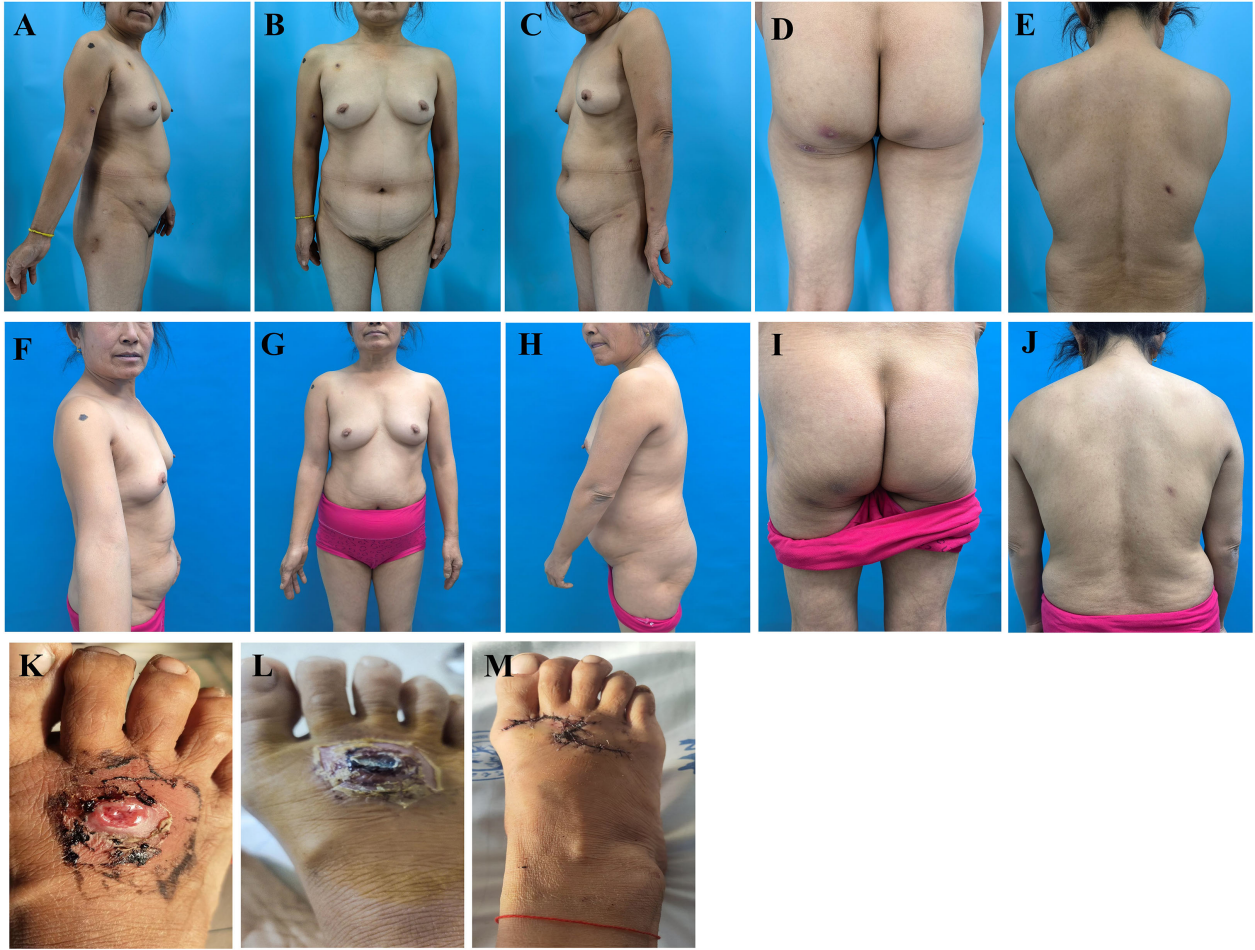
Comparison images of the patient’s skin lesions on the trunk, limbs, buttocks, and dorsum of the right foot before and after treatment (foot photos provided by the patient). **(A–E)** Before treatment, the patient exhibited scattered nodules, ulcers, necrosis, and scabs on the trunk and limbs. **(F–J)** After treatment, all nodules resolved completely, with residual scars and focal pigmentation noted in some skin areas. **(K, L)** In the early disease phase, a large deep ulcer developed on the right dorsal foot, followed by exudation and scab formation. **(M)** The patient achieved recovery following surgical intervention with flap repair at Hospital A.

Physical examination revealed no palpable enlarged superficial lymph nodes throughout the body and no hepatosplenomegaly. Scattered dark red nodules, ranging in size from 0.5 cm to 1.0 cm, were observed on the chest, back, limbs, and buttocks. These nodules were partially covered with scales, and some areas exhibited central ulcers with necrosis and crusting. The lesions had well-defined red margins, a firm texture, good mobility, and were non-tender upon palpation. Postoperative scars were noted on the back, the right forearm, and the dorsum of the right foot (**Figures 1A–E**). Additionally, the patient retained pre- and post-surgical photographs of the skin on the dorsum of the right foot. Prior to surgery, a large ulcer measuring approximately 3.0 cm × 4.0 cm was present on the right foot, characterized by a raised edge resembling a riverbank and a central depression akin to a volcanic crater. The central region exhibited serosanguineous exudation, while the lesion’s borders were erythematous, edematous, and poorly demarcated (**Figures 1K, L**). After treatment, the nodules on the trunk, buttocks, and extremities completely regressed, leaving only minimal post-inflammatory (**Figures 1F–J**). Following surgery, a 7 cm incision was made on the dorsum of the right foot, which was sutured with a skin flap (**Figure 1M**).

**Figure 2:**
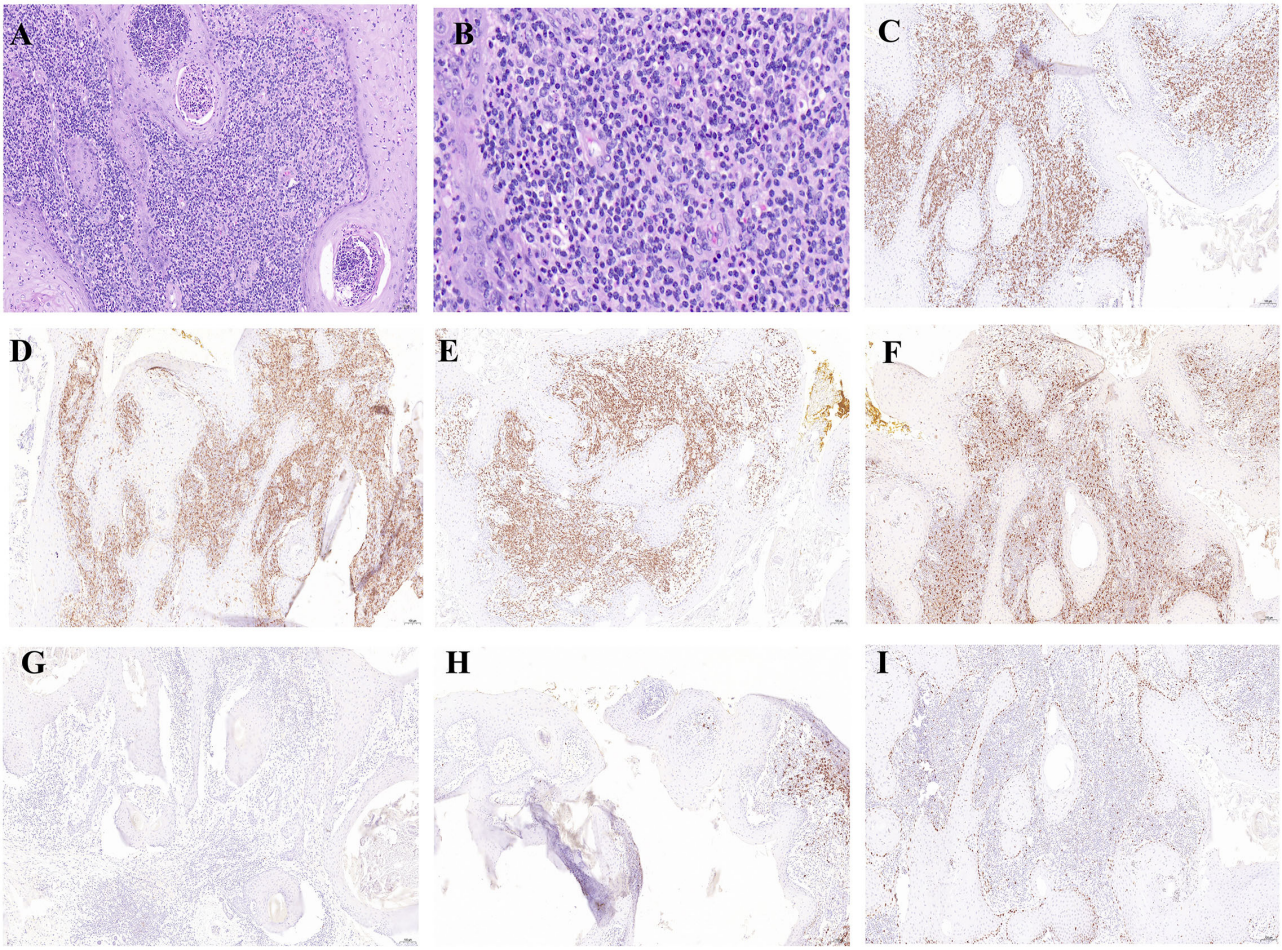
Histological and immunohistochemical examination of the large ulcer on the dorsum of the right foot. **(A)** Histopathological examination of the foot lesion revealed cup-shaped epidermal proliferation with a central keratin-filled crater, well-differentiated squamous cells, and a mixed inflammatory infiltrate in the dermis, findings consistent with KA.(×100). **(B)** High-power magnification revealed scattered atypical lymphocytes (×400). **(C–I)** Immunohistochemistry staining for CD8 (+), CD4 (diffuse+), CD3 (diffuse+), CD68 (+), CD30 (–), CD20 (focal+), Ki-67 positivity of approximately 5% (×100).

The histopathological examination of a lesion from the dorsum of the right foot (Hospital A, December 8, 2023) demonstrated characteristic epidermal changes including PEH. The dermis exhibited significant inflammatory infiltration composed of dense lymphocytic aggregates admixed with histiocyte-like cells, accompanied by atypical keratinocyte proliferation. Notably, scattered atypical lymphocytes displaying hyperchromatic nuclei, prominent nucleoli and abundant cytoplasm were observed throughout the dermal layers. The above pathological manifestations suggest KA (**Figures 2A, B**). To investigate potential associations between the patient’s prior “foot KA” presentation and subsequent LyP diagnosis, we communicated with the patient and utilized paraffin-embedded blocks preserved after surgery at Hospital A for immunohistochemical analysis. The results showed CD8 (+), CD4 (diffuse+), CD3 (diffuse+), CD68 (+), CD30 (–), CD20 (focal+), and Ki-67 positivity expression rates was approximately 5% (**Figures 2C–I**).

**Figure 3:**
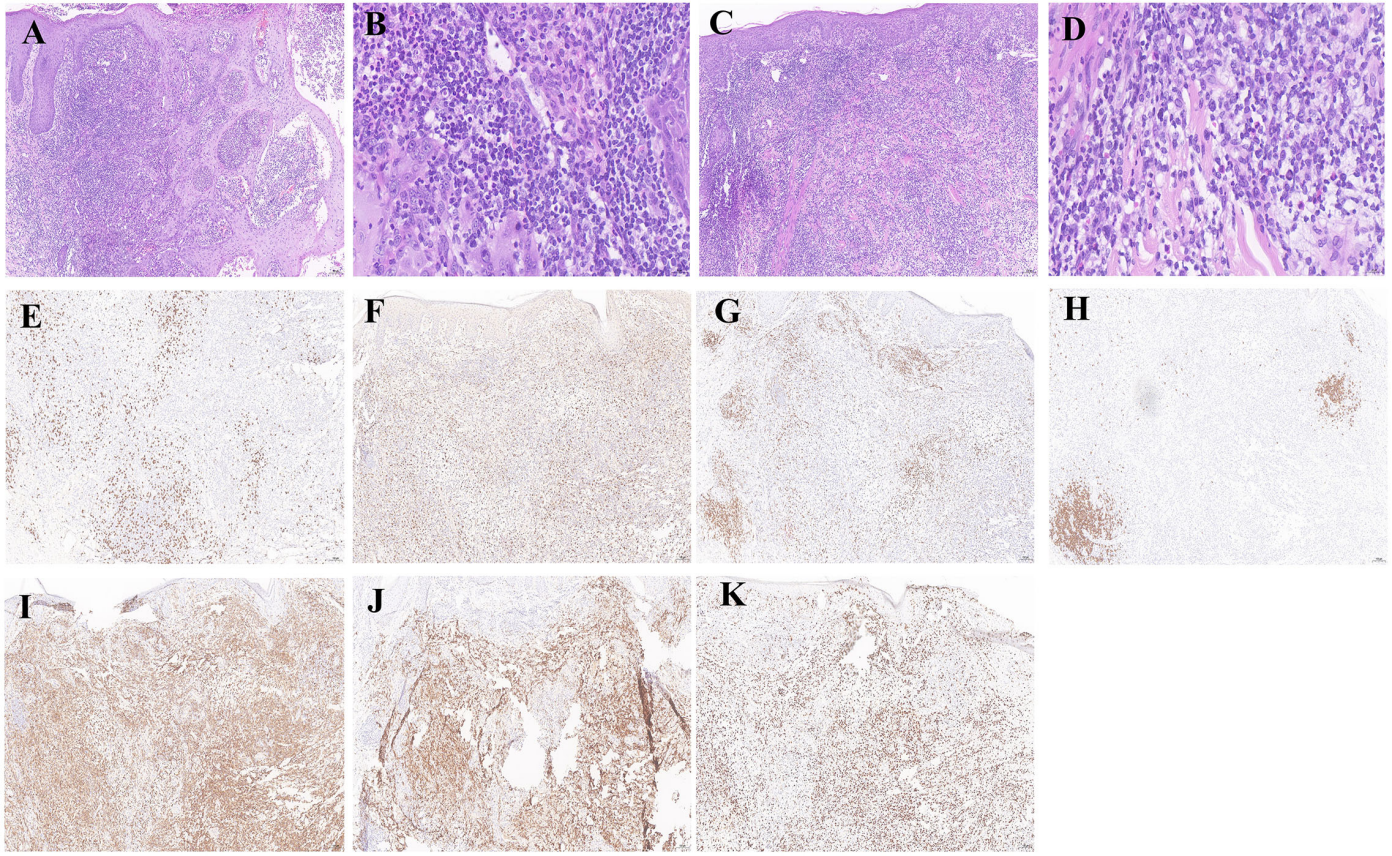
Histological and immunohistochemical examinations of nodular tissues in the right forearm and back. **(A, C)** Histopathological examination of the right forearm and back revealed atypical epidermal hyperplasia, extensive wedge-shaped lymphocytic infiltration in the dermis with admixed neutrophils, eosinophils, and histiocytes, and larger atypical lymphocytes, findings consistent with LyP (×100). **(B, D)** High-power magnification revealed prominent infiltration of atypical lymphocytes in the right forearm and back tissues (×400). **(E–K)** Immunohistochemical staining for CD8 (scattered+) was observed, with CD68 (diffuse+), CD3 (+), CD20 (focal+), CD4 (diffuse+), and CD30 (diffuse+), and Ki-67 positivity of approximately 50% (×100).

The dermatopathology report of the lesion from the right forearm (Hospital B, December 20, 2023) demonstrated moderate to severe atypical hyperplasia of the epidermis. A wedge-shaped infiltration of atypical lymphoid cells was observed in the dermis. These cells exhibited medium to large sizes with twisted, hyperchromatic nuclei and visible mitotic figures. Additionally, a mixed inflammatory infiltrate comprising small lymphocytes, histiocytes, neutrophils, and eosinophils was noted. PAS staining did not reveal any positive spores or hyphae (**Figures 3A, C**). The histopathological examination of the lesion from the back revealed the following characteristics. The infiltrating cells were identified as atypical lymphocytes with markedly pleomorphic nuclei that were hyperchromatic or vacuolated, prominent nucleoli, and abundant cytoplasm. The infiltrating cells also include neutrophils, eosinophils, lymphocytes, and histiocytes. (**Figures 3B, D**). On February 4, 2024, paraffin blocks were obtained from Hospital B for resection and immunohistochemical analysis: CD8 (scattered+), CD68 (diffuse+), CD3 (+), CD20 (focal+), CD4 (diffuse+), CD30 (diffuse+), Ki-67 positivity expression rates for about 50%. Findings were consistent with LyP (**Figures 3E–K**).

**Figure 4:**
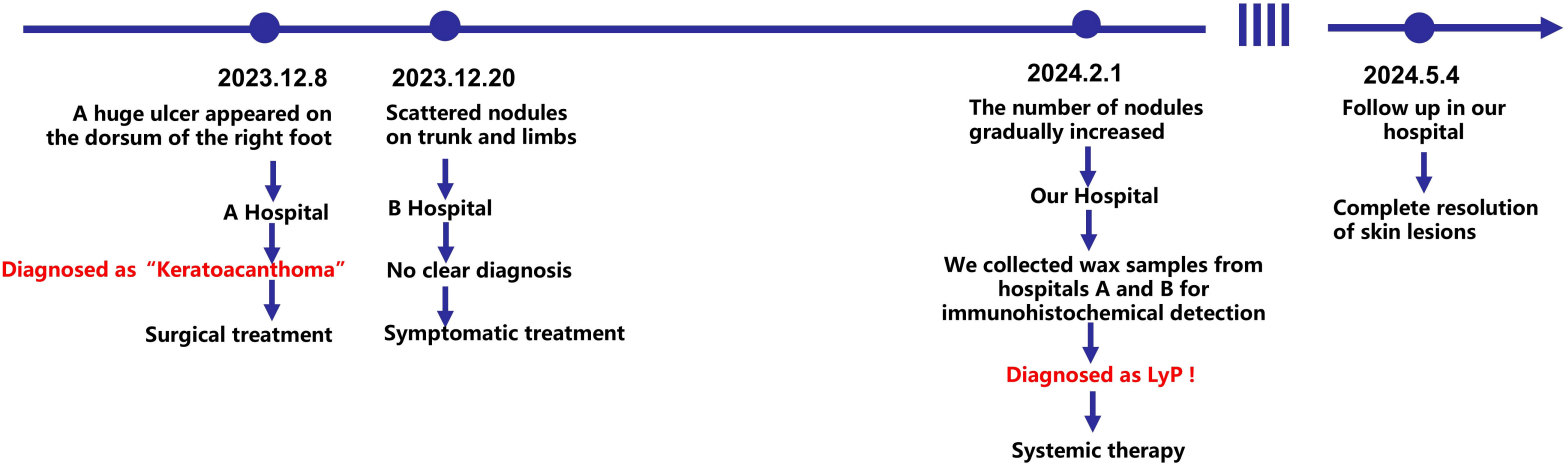
Schematic illustration of the clinical course of the patient.

The patient was definitively diagnosed with LyP Type A. Comprehensive laboratory and ancillary examinations were performed, revealing no abnormalities in routine blood tests, urine analysis, myocardial enzymes, liver function, renal function, tumor markers, abdominal ultrasound, cardiac ultrasound and thoracic CT. The treatment regimen consisted of methotrexate (2.5 mg orally every 12 hours for three consecutive doses within one week), folic acid tablets (5 mg orally once daily), and interferon-α2b (IFN-α2b, 3 million units administered via intramuscular injection daily for 15 consecutive days). Subsequently, the IFN-α2b dosing schedule was adjusted to 3 million units every 5 days for a total of three injections, then modified to every 10 days for another three injections, and finally adjusted to every 15 days for two injections before discontinuation. Additionally, the patient applied a 5% imiquimod cream topically once daily. The patient was advised to undergo monthly follow-up evaluations, including routine blood tests, urine analysis, liver and renal function tests, all of which have remained normal to date. Upon re-evaluation after three months of treatment, the nodules on the trunk, buttocks, and limbs had completely resolved, leaving only minimal post-inflammatory hyperpigmentation. No new nodules emerged during the treatment period. The patient expressed satisfaction with the results. The clinical course of the patient is illustrated in **Figure 4**.

## Discussion

LyP is a rare, chronic, recurrent, and self-healing low-grade malignant cutaneous indolent lymphoma (1, 2). The etiology and pathogenesis of this disease remain unclear but may involve associations with paramyxovirus, human T-cell leukemia/lymphoma virus, Epstein-Barr virus, mosquito bites, topical drug irritation, and infections (6). The male-to-female ratio of LyP is approximately 2:1, and LyP can occur in individuals of all age groups (7–9). Typical clinical manifestations include asymptomatic erythema, papules, and nodules that appear in crops, predominantly on the hands, feet, and trunk. These lesions are usually several to a dozen in number and symmetrically distributed, with a diameter of less than 2 cm. They may coalesce into plaques and gradually evolve into centrally necrotic and hemorrhagic papules, which can spontaneously regress and leave atrophic scars on the skin surface after healing. Skin lesions in patients can resolve within several weeks to months, while the overall disease course can persist for 5-10 years or longer (10).

Based on histopathological changes and genetic testing, the 5th edition of the WHO Classification of Haematopoietic and Lymphoid Tumours categorizes LyP into types A, B, C, D, E, and F, as well as several subtypes (11). Types A, B, and C are considered the most classical forms. Among these, LyP Type A accounts for approximately 80% of the cases, which is characterized by the infiltration of large anaplastic cells with markedly pleomorphic nuclei that are hyperchromatic or vacuolated, prominent nucleoli and abundant cytoplasm. The infiltrates also include lymphocytes, histiocytes, eosinophils, and neutrophils. LyP Type B is characterized by wedge-shaped or band-like epidermotropic infiltration of CD4-positive small-to medium-sized pleomorphic cells in the dermis, with tumor cells resembling the gyrate lymphocytes of mycosis fungoides. LyP Type C represents a borderline type that is histologically similar to anaplastic large-cell lymphoma (12). In recent years, some scholars have proposed the classification of LyP Type D, which is characterized by a significant epidermotropic CD8+ T-lymphocyte infiltrate. More than half of patients with LyP exhibit clonal T-cell gene rearrangements (13). Special types of LyP include folliculotropic, eccrine, and 6p25.3 chromosomal rearrangement types (14–17).

Additionally, a special type of LyP is accompanied by PEH, and some skin lesions are pathologically similar to KA, KA is a subtype of SCC with a tendency toward spontaneous regression. Dermatopathologists refer to the lesion as *squamous cell carcinoma, keratoacanthoma type* (SCC–KA type) (18, 19). A few case reports have also described LyP associated with SCC-like or KA-like skin changes (3–5, 20). Nevertheless, these so-called KA-like or SCC-like changes are considered to reflect reactive hyperplasia of the epidermis secondary to LyP rather than actual KA or SCC. Several researchers have endeavored to clarify the relationship between LyP and PEH from a pathological perspective. Courville proposed that epidermal growth factor (EGF) is associated with epidermal hyperplasia linked to lymphoproliferative disease, which likely involves multiple mediators, including EGF, transforming growth factor-alpha (TGF-α), and epidermal growth factor receptor (EGFR). Compared to T-cell lymphoma without PEH, skin T-cell lymphoma accompanied by PEH exhibited stronger expression of EGF and TGF-α on T cells and EGFR on epidermal cells. However, subsequent studies did not yield the same findings (5), suggesting that the pathogenesis of epidermal hyperplasia might be more complex than currently appreciated, warranting further exploration.

LyP can be differentiated from other diseases by integrating clinical presentation and histopathological features (21, 22). Acute Lichenoid Pustular Keratosis (ALPK) shares similar papulonodular lesions with LyP clinically. Histopathologically, ALPK is characterized by a CD8+ T-cell-dominant infiltrate with epidermal pustule formation, whereas CD30+ anaplastic large cells—pathognomonic for LyP—are absent. Several key factors led us to favor the diagnosis of LyP over C-ALCL despite the diffuse CD30 positivity and overlapping clinical manifestations. Clinically, LyP shows papules or nodules, 1-2 cm in diameter, red or purplish red, common on the trunk and limbs. C-ALCL features larger nodules/masses (over 2 cm), darker, firmer, occurring on the head, neck, trunk, and limbs, either single or multiple. Pathologically, our case’s infiltrate was a mixed cell population (lymphocytes, histiocytes, eosinophils) with only a small proportion of large atypical cells, this mixed-cell infiltrate pattern is more consistent with LyP. In contrast, C-ALCL has at least 75% of the tumor cells express the CD30 antigen. C-ALCL infiltrates deeper into the fat layer, while LyP Type A often shows a wedge-shaped infiltration pattern, consistent with our case. Thus, tumor invasion depth and the wedge-shaped structure in the dermis help distinguish C-ALCL (22, 23). Furthermore, LyP must also be distinguished from conditions such as lymphomatoid insect bite reactions and Hodgkin lymphoma. Based on the clinical and pathological manifestations, the disease can be distinguished from similar conditions.

CD30 is one of the core markers for the diagnosis, classification, and targeted therapy of lymphoma, and is used to assist in the differentiation of CD30-positive lymphomas such as C-ALCL and LyP. However, subsequent research has revealed its expression in various inflammatory and infectious skin diseases as well as non-lymphoma tumors, including viral, bacterial, superficial fungal and mycobacterial infections, allergic contact dermatitis, drug-induced reactions, insect bite reactions and scabies (24–27). A definitive diagnosis of LyP typically definitive on the positivity rate of CD30, ranging from 25% to 90% (28). While CD30+ cells are considered a diagnostic hallmark of CD30+ lymphoproliferative disorders, they are not absolute indicators. In some cases of LyP or LyP with KA-like changes, CD30+ cells may only be sporadically detected and in certain instances, CD30+ cells have not been documented (5, 29). Some reports in the literature have indicated that a small number of lyP cases are accompanied by CD30 negative expression (30–32). There is an overlap between the reactive CD30+ cells observed in KA and the KA-like reactive changes observed in LyP (29, 33, 34). This case report corroborates the aforementioned findings.

In this case, the initial diagnosis of a gaint ulcer on the dorsum of the right foot was diagnosed as KA This may be attributed to the pathology reporters at Hospital A focusing primarily on the abnormal proliferation of keratinocytes while overlooking the infiltration of abnormal lymphocytes. Emerging evidence suggests histological overlap between KA and LyP, potentially driven by shared triggers such as infections, or inflammatory stimuli that concurrently activate keratinocyte hyperplasia (35). However, LyP is accompanied by atypical lymphocytes displaying hyperchromatic nuclei, prominent nucleoli and abundant cytoplasm. The expression of CD30 was negative in the patient’s initial lesion, whereas subsequent scattered nodules on the trunk and limbs exhibited positive CD30 expression. This may signify dynamic disease progression, where LyP manifestations evolve through distinct histopathological phases. Case reports published by Guitart (20), original research by Fernandez-Flores (36), and studies published by Scarisbrick all align with our perspectives (5).

LyP is an indolent disease characterized by self-healing properties. Treatment strategies are primarily focused on controlling rash dissemination and reducing the frequency of recurrence. The 5-year survival rate is nearly 100%, underscoring the importance of regular follow-up and symptomatic management. For patients presenting with clinical symptoms, treatments such as phototherapy, including psoralen plus ultraviolet A (PUVA), narrowband ultraviolet A (UVA), and 308 nm excimer laser, can be administered based on individual circumstances (37). Local or systemic application of medications such as glucocorticoids, methotrexate (MTX), and antibiotics may also be considered. Notably, MTX suppresses lymphocyte proliferation and effectively counteracts lymphoproliferation associated with LyP (38). Recent studies indicate that combining MTX and interferon-alpha (IFN-α) is effective in treating LyP (39). In this case, treatment with MTX combined with IFN-α2b led to complete resolution of the rash, which has not recurred to date.

## Conclusion

In summary, a giant ulcer exhibiting PEH in histopathology may indicate LyP. Careful assessment for atypical lymphocytic infiltration and further immunohistochemical evaluation are essential for accurate diagnosis. When single clinical or histopathological findings are insufficient to provide a comprehensive understanding of the disease, thorough evaluation and dynamic monitoring are critical for diagnosing and managing complex cases. By presenting this case and reviewing the literature on LyP, this case report aims to enhance clinicians’ awareness of the rare manifestations of LyP.

## Data Availability

The original contributions presented in the study are included in the article/**Supplementary Material**. Further inquiries can be directed to the corresponding authors.
