# Supplementary material for: Rare type A lymphomatoid papulosis initially presenting as a giant ulcer: a case report and literature review
Source: Front Immunol. 2025 Jul 1;16:1606027. doi: 10.3389/fimmu.2025.1606027 (PMC12259569; doi:10.3389/fimmu.2025.1606027)
Supplement: Supplementary file 1 [file DataSheet1.pdf]

CARE
case report guidelines

CARE Checklist of information to include when writing a case report

(cc) BY-NC-ND

Topic	Item	Checklist item description	Reported on Line
Title	1	The diagnosis or intervention of primary focus followed by the words "case report"	Title
Key Words	2	2 to 5 key words that identify diagnoses or interventions in this case report, including "case report"	Key woeds
Abstract (no references)	3a	Introduction: What is unique about this case and what does it add to the scientific literature?	the 3rd sentences of the case presentation in abstract
	3b	Mainsymptoms and/or important clinical findings	the first and the second sentences of the case presentation in abstract
	3c	The main diagnoses, therapeutic interventions, and outcomes	the 4th,5th,6th sentences of the case presentation in abstract
	3d	Conclusion—What is the main "take-away" lesson(s) from this case?	conclusion in abstract
Introduction	4	One or two paragraphs summarizing why this case is unique (**may include references**)	introduction
Patient Information	5a	De-identified patient specific information.	the first sentence of the case presentation
	5b	Primary concerns and symptoms of the patient.	the first paragraph of the case presentation
	5c	Medical, family, and psycho-social history including relevant genetic information	the last sentence of the of the first paragraph in case presentation
	5d	Relevant past interventions with outcomes	the 5th paragraph of the case presentation
Clinical Findings	6	Describe significant physical examination (PE) and important clinical findings.	The second paragraph of the case presentation
Timeline	7	Historical and current information from this episode of care organized as a timeline	the last sentence of the last paragraph in case presentation
Diagnostic Assessment	8a	Diagnostic testing (such as PE, laboratory testing, imaging, surveys).	the 2nd,the 3rd and the 4th paragraphs of the case presentation
	8b	Diagnostic challenges (such as access to testing, financial, or cultural)	the 2nd sentence of the of the 3rd paragraph in case presentation
	8c	Diagnosis (including other diagnoses considered)	the first sentence of the of the 5th paragraph in case present
	8d	Prognosis (such as staging in oncology) where applicable	the last paragraph in case presentation
Therapeutic Intervention	9a	Types of therapeutic intervention (such as pharmacologic, surgical, preventive, self-care)	the last paragraph in case presentation
	9b	Administration of therapeutic intervention (such as dosage, strength, duration)	the last paragraph in case presentation
	9c	Changes in therapeutic intervention (with rationale)	the 3rd,the 4th and the 5th sentences of the of the last paragraph in case presentation
Follow-up and Outcomes	10a	Clinician and patient-assessed outcomes (if available)	the6th sentence of the of the last paragraph in case presentation
	10b	Important follow-up diagnostic and other test results	NA
	10c	Intervention adherence and tolerability (How was this assessed?)	the 7th and 8th sentence of the of the last paragraph in case presentation
	10d	Adverse and unanticipated events	NA
Discussion	11a	A scientific discussion of the strengths AND limitations associated with this case report	The 3rd and the 4th paragraphs of the discussion
	11b	Discussion of the relevant medical literature **with references**.	The first, second, and the third paragraphs of the discussion
	11c	The scientific rationale for any conclusions (including assessment of possible causes)	The first, second, third and the fourth paragraphs of the discussion
	11d	The primary "take-away" lessons of this case report (without references) in a one paragraph conclusion	the conclusion
Patient Perspective	12	The patient should share their perspective in one to two paragraphs on the treatment(s) they received	the seventh sentence of the last paragraph in case presentation
Informed Consent	13	Did the patient give informed consent? Please provide if requested	Yes ☑ No ☐
